# Supplementary material for: Cultural, sociopolitical, environmental and built assets supporting health and well-being in Torres Strait Island communities: protocol for a scoping review
Source: BMJ Open. 2023 Dec 8;13(12):e077229. doi: 10.1136/bmjopen-2023-077229 (PMC10729014; doi:10.1136/bmjopen-2023-077229)
Supplement: Supplementary data [file bmjopen-2023-077229supp001.pdf]

Appendix A

Draft search strategy

14 April 2023

Informit

Databases (Health Collection, Humanities and Social Science Collection; Indigenous Collection; Aboriginal and Torres Strait Islander Health Bibliography (ATSIHealth); Indigenous Studies Bibliography (AIATSIS)

Torres Strait Island* AND community AND Environment AND (plan OR policy OR report) (all fields)

Webb T, *et al. BMJ Open* 2023; 13:e077229. doi: 10.1136/bmjopen-2023-077229
