# Supplementary material for: Cultural, sociopolitical, environmental and built assets supporting health and well-being in Torres Strait Island communities: protocol for a scoping review
Source: BMJ Open. 2023 Dec 8;13(12):e077229. doi: 10.1136/bmjopen-2023-077229 (PMC10729014; doi:10.1136/bmjopen-2023-077229)
Supplement: Author's manuscript [file bmjopen-2023-077229.draft_revisions.pdf]

BMJ Open is committed to open peer review. As part of this commitment we make the peer review history of every article we publish publicly available.

When an article is published we post the peer reviewers' comments and the authors' responses online. We also post the versions of the paper that were used during peer review. These are the versions that the peer review comments apply to.

The versions of the paper that follow are the versions that were submitted during the peer review process. They are not the versions of record or the final published versions. They should not be cited or distributed as the published version of this manuscript.

BMJ Open is an open access journal and the full, final, typeset and author-corrected version of record of the manuscript is available on our site with no access controls, subscription charges or pay-per-view fees (http://bmjopen.bmj.com).

If you have any questions on BMJ Open's open peer review process please email info.bmjopen@bmj.com

BMJ Open

Cultural, socio-political, environmental, and built assets supporting health and wellbeing in Torres Strait Island communities: Protocol for a scoping review

Journal:	*BMJ Open*
Manuscript ID	bmjopen-2023-077229
Article Type:	Protocol
Date Submitted by the Author:	29-Jun-2023

Complete List of Authors:	Webb, Torres; James Cook University College of Medicine and Dentistry<br>Meldrum, Kathryn; James Cook University College of Medicine and Dentistry<br>Kilburn, Melissa; James Cook University College of Medicine and Dentistry<br>Wallace, Valda; James Cook University College of Medicine and Dentistry, College of Medicine and Dentistry<br>Russell, Sarah; James Cook University College of Medicine and Dentistry, College of Medicine and Dentistry<br>Quigley, Rachel; James Cook University College of Medicine and Dentistry, College of Medicine and Dentistry<br>Strivens, Edward; James Cook University College of Medicine and Dentistry; Queensland Health, Cairns and Hinterland Hospital and Health Service

Keywords:	PUBLIC HEALTH, Risk Factors, Systematic Review, Geographical mapping < TROPICAL MEDICINE

SCHOLARONE™
Manuscripts

Cultural, socio-political, environmental, and built assets supporting health and wellbeing in Torres Strait Island communities: Protocol for a scoping review

Torres Webb, College of Medicine and Dentistry and Australian Institute of Health and Tropical Medicine, Building E1.003a, James Cook University, PO Box 6811, Cairns, Queensland, Australia. [1.]*

Kathryn Meldrum, College of Medicine and Dentistry and Australian Institute of Health and Tropical Medicine. Kathryn.meldrum@jcu.edu.au (corresponding author)*

Melissa Kilburn, College of Medicine and Dentistry and Australian Institute of Health and Tropical Medicine, James Cook University, Cairns, Queensland, Australia

Valda Wallace, College of Medicine and Dentistry and Australian Institute of Health and Tropical Medicine, James Cook University, Cairns, Queensland, Australia.[1.]

Sarah Russell, College of Medicine and Dentistry and Australian Institute of Health and Tropical Medicine, James Cook University, Cairns, Queensland, Australia.

Rachel Quigley, College of Medicine and Dentistry and Australian Institute of Health and Tropical Medicine, James Cook University, Cairns, Queensland, Australia.

Edward Strivens, College of Medicine and Dentistry and Australian Institute of Health and Tropical Medicine, James Cook University, Cairns, Queensland, Australia. Queensland Health, Cairns and Hinterland Hospital and Health Service, Cairns, Queensland, Australia.

[1.]This person identifies as a First Nations Australian

*Joint first authors

Word count - 2209

For peer review only - http://bmjopen.bmj.com/site/about/guidelines.xhtml

ABSTRACT

Introduction Risks to an individual's health should be considered alongside the environmental, socio-cultural and socio-political context(s) in which they live. Environmental mapping is an approach to identifying enablers and barriers to health within a community. The Indigenous Indicator Classification System (IICS) framework has been used to map the environment in Australian Indigenous communities. The IICS is a four-level nested hierarchical framework with subject groups including culture, socio-political and built at the top of the hierarchy and indicators at the bottom. The objective of this scoping review is to map the cultural, socio-political, environmental, and built assets that support health and wellbeing that exist in each Torres Strait Island community.

Methods and analysis This review will be conducted according the JBI method for scoping reviews. It will include sources that identify cultural, socio-political, environmental, and built assets that support health and wellbeing that exist in each Torres Strait Island community. Databases to be searched include: Informit; Scopus; Web of Science; HealthInfoNet, BioOne Complete and Green File. Sources of unpublished and grey literature will be located using Google and Google Scholar. Searches will be limited to the English language and literature published since January 2018 to ensure that the assets mapped reflect current conditions on each island. Data that answers the research question will be extracted from sources and recorded in an adaptation of the IICS. Quantitative analysis of the data will include summing each asset for individual islands and their associated clusters. Data will be presented graphically, diagrammatically, or in tabular form depending on what approach best conveys its meaning.

Ethics and dissemination The Far North Queensland Human Research Ethics Committee (reference HREC/2022/QCH/88155-1624) has approved this study. Dissemination of the review's findings will be led by Torres Strait Islander members of the research team through conferences and peer-reviewed publications.

For peer review only

For peer review only - http://bmjopen.bmj.com/site/about/guidelines.xhtml

STRENGTHS AND LIMITATIONS OF THIS STUDY

- This will be the first scoping review that maps available sources to identify cultural, socio-political, environmental, and built assets that support health and wellbeing that exist in each Torres Strait Island community.
- The scoping review will be conducted according to the method prescribed by the Joanna Briggs Institute.
- The findings will be used to support the next phase of the project, community yarns about strengths and barriers to health and wellbeing on Torres Strait Island communities.
- Data collection will be limited to sources that are able to be located through desk-based searches which may mean that some cultural, socio-political, environmental, and built assets will not be recorded.

INTRODUCTION

Mapping the environment is an approach to identifying barriers to health and wellbeing within a community [1-3]. Environmental mapping is a robust approach because, as Rose [4] identified, the risk to an individual's health should be considered alongside the environmental, socio-cultural and socio-political context(s) in which they live. One approach to environmental mapping in an Australian Indigenous community in Western Australia focused mainly on barriers to health attributable to the environment [1]. This focus failed to recognise that the environment can also be a protective factor [5], especially in Australian Indigenous communities [2, 6, 7]. Le Gal and colleagues [8] supported a broader conceptualisation of health, inclusive of the environment, for Indigenous Australians. The rationale for broader conceptualisation builds on the previous work of Daniel and his colleagues [2]. Their review [2] argued that the context and composition of the environment act together as risks for health and wellbeing in Australian Indigenous communities. The authors defined context as the "properties of places" (p. 330) and composition as "collective attributes of people in a place" (p. 330)

For peer review only

For peer review only - http://bmjopen.bmj.com/site/about/guidelines.xhtml

[2]. Environmental context and composition coalesce to interact with an individual's genetic, behavioural and stress axes to predispose them to chronic disease.

The Indigenous Indicator Classification System as an approach to environmental mapping

Early work by Marks and colleagues [9, 10] proposed the Indigenous Indicator Classification System (IICS) as a framework that could be used to map the "distinct historical, social and cultural contexts of Indigenous communities" (p. 93). The IICS was developed in 2006 with input from Indigenous peoples from Canada, Aotearoa/New Zealand and Australia [2] and validated in 2009 [10].

The IICS is a hierarchical four-level system with subject groups at the highest level. At the time of development, subject groups consisted of culture, socio-demographic, socio-political, socio-economic, built, and natural environment, psychosocial, and social organisations. For example, cultural assets include the background and history of peoples and the traditional activities and cultural responsibilities that they undertake [9]. Socio-political assets include, but are not limited to, community economic resources, Indigenous self-government and autonomy and labour market and working conditions. Whereas, built assets include but are not limited to the natural environment, housing, and transportation. The subject groups are the broadest classification level to which each indicator group or goal dimension is allocated to [9]. Domains are the broadest of the nested hierarchical categories [9] and identify the focus of the goal dimension and/or indicator group [11]. Goal dimensions describe the parameters for classifying indicators and could be used to identify potential targets for health policies and/or programs [9]. Consequently, in the IICS they often describe a desirable community outcome. For example, community business and economic development [3]. Indicators are measurable information [12] able to be found in documents, grey literature or through community conversations. In Australia, health indicators, such as those used in the IICS, describe particular elements of health or aspects of performance [13]. The link(s) between domains and

For peer review only - http://bmjopen.bmj.com/site/about/guidelines.xhtml

indicators are important for explaining and providing a platform for investigating the complexity of influences on Indigenous peoples' health [9] and wellbeing. Figure 1 below illustrates the IICS using one example of the built and natural environment.

The context of the protocol and scoping review

The setting for this study is the Torres Strait (Zenadth Kes) situated between the tip of the eastern Australian mainland and Papua New Guinea. There are over 200 islands in the Torres Strait located in a vast geographic area of over 44,000 square kilometres. However, only 17 islands are permanently inhabited [14]. In 2016, approximately 7403 people lived on the islands of the Torres Strait, 86.6% of whom identified as Australian First Nations peoples [15].

Rationale and aims

The context of this scoping review is a broader project funded by the Australian National Health and Medical Research Council (grant number MRF2016931) designed to address significant gaps in existing knowledge and develop an understanding of key assets for a strengths-based approach to targeting diet and activity components of chronic disease risk for First Nations peoples living in the Torres Strait. The broader project is designed to:

- Identify the strengths and barriers to good health in Torres Strait communities;
- Identify dietary and activity behaviours of Australian First Nations peoples living in the Torres Strait; and,
- Use this information to develop community-led chronic disease prevention interventions at an individual and service provider level.

The aim of the scoping review that this protocol refers to is to addresses dot point 1) (above) initially by mapping the cultural, socio-political, natural, and built environment of each of the inhabited Islands of the Torres Strait. The research question guiding this scoping review is: What cultural,

For peer review only - http://bmjopen.bmj.com/site/about/guidelines.xhtml

socio-political, environmental, and built assets that support health and wellbeing exist in each Torres Strait Island community?

METHODS AND ANALYSIS

The proposed scoping review will be conducted in accordance with the JBI methodology for scoping reviews [16]. Both this protocol and subsequent scoping review will be reported using the Preferred Reporting Items for Systematic Reviews and Meta-analyses Extension for Scoping Review (PRISMA-ScR) guidelines [17].

Inclusion criteria

In line with the JBI methodology the Participant, Concept, Context (PCC) framework will be used to guide inclusion criteria and search strategy for the scoping review.

Participants

The scoping review will include sources that identify and/or describe any cultural, socio-political, natural, and built assets (hereafter referred to collectively as assets) that support health and wellbeing in Torres Strait Island communities which may include people and programs.

Concept

The concepts to be examined in the scoping review will include assets that support health and wellbeing in Torres Strait Island communities. Specific data that will be extracted from included sources will be:

- The island and/or community where the asset is located;
- Identification of the type of asset.

Sources that will be excluded from the data set includes any secondary information about assets located on any Torres Strait Island community. The rationale for excluding secondary sources is that

For peer review only - http://bmjopen.bmj.com/site/about/guidelines.xhtml

it may be out of date or may not be accurate. In addition, the information must describe actual assets not aspirational ones that may be detailed in regional plans.

Context

Any source that details the assets of Torres Strait Island communities will be considered for inclusion in the scoping review. The sources may be obtained from database searches or from grey literature located on key regional governance websites such as Torres Strait Regional Authority, Torres Strait Island Regional Council, Gur A Baradharaw Kod Torres Strait Sea and Land Council, and Torres Shire Council.

Types of sources

Relevant sources that have been peer-reviewed or have been obtained from grey literature will be included in the data set. Sources may include annual reports, strategic, corporate, operational or implementation plans, evaluations, planning documents and published literature. Critical reviews, literature reviews and systematic reviews will not be included in the data set because they are not primary data sources. However, their reference lists will be reviewed for relevant primary data sources.

Inclusion criteria in order of priority will be:

1. Information that identifies assets available/present in a Torres Strait Island community.
2. Uses primary data sources.
3. Has been published since 2018.

The scoping review is registered with The Open Science Framework (https://osf.io/2wv8c).

Search strategy

The search strategy will aim to locate published and unpublished sources. A three-step search strategy will be utilised in this review. First, an initial limited search of Web of Science and Informit was

For peer review only - http://bmjopen.bmj.com/site/about/guidelines.xhtml

undertaken to identify relevant literature. The text words contained in the titles and abstracts of relevant sources, and the index terms used to describe them were used to develop a full search strategy tailored to each information source. For example, a pilot search strategy is illustrated in the online supplement materials *(Appendix A*). Step one was supported by a subject-specific liaison librarian. The reference lists of all included sources of evidence will be screened for additional sources. Additionally, reference lists of systematic reviews on the same or similar topic will be scrutinised for appropriate papers, reports, or other data sources.

Published or unpublished reports or other data sources in the English language and available since the year 2018 to present will be included in the data set. Sources published since 2018 will be used because they are more likely to identify assets currently present in Torres Strait Island communities.

Databases to be searched include:

- Informit;
- Scopus;
- Web of Science;
- BioOne Complete;
- HealthInfoNet;
- Green File.

Unpublished and grey literature will be identified using Google, Google Scholar and website searches.

Source of evidence selection

At the commencement of step two all identified citations will be collated and uploaded into EndNote (Version 20.1) [18] and data will be subsequently managed according to the method proposed by Peters [19]. A pilot test will then be conducted as described by Peters and colleagues [16], following which, titles and abstracts will be screened by two independent reviewers for assessment against the

For peer review only - http://bmjopen.bmj.com/site/about/guidelines.xhtml

inclusion criteria for the review. Potentially relevant sources will be retrieved in full and subsequently assessed against the inclusion criteria by two independent reviewers. Reasons for exclusion of full text sources of evidence that do not meet the inclusion criteria will be recorded and reported in the scoping review. Any disagreements that arise between the reviewers at each stage of the selection process will be resolved through discussion. Where agreement cannot be achieved through discussion an additional reviewer/s will review the source. The results of the search and the study inclusion process will be reported in full in the final scoping review and presented in a Preferred Reporting Items for Systematic Reviews and Meta-analyses extension for scoping review (PRISMA-ScR) flow diagram [20].

Data extraction

Data will be extracted from sources included in the scoping review by two independent reviewers using the data extraction tool, an adaptation of the IICS [3, 9, 10]. Data extracted from sources will include specific information about the participants, concept, and context. Any further adaptations to the IICS made during data extraction will be detailed in the scoping review. Any disagreements will be managed in the manner previously discussed. If appropriate, source authors will be contacted to request missing or additional information, where required. In addition, key informants in regional governance bodies will be contacted to confirm source information when necessary.

Data analysis and presentation

Data will be analysed quantitatively to identify how it is distributed geographically across the Torres Strait Islands. Assets used will be categorised into the overarching groups:

- Cultural;
- Socio-political;
- Environmental, and,
- Built.

For peer review only - http://bmjopen.bmj.com/site/about/guidelines.xhtml

Once an asset has been categorised into a group it will subsequently be allocated into an indicator group by referring to the relevant indicator statement. The presence of an asset will be recorded by typing a '1' into the relevant column in the database. Once data collection is complete, asset sums will be calculated for each indicator, for each Island and Island group.

Data will be presented graphically, diagrammatically or in tabular form depending on what approach that best represents the data. For example, using Geographic Information System (GIS) mapping software may be used to indicate what assets are available on each island as this approach potentially conveys greater meaning than tabulating it. A narrative summary will accompany the tabulated and/or charted results and will describe how the results relate to the review's objective and question.

For peer review only

Patient and public involvement

As previously indicated this study is phase one of a broader four phase study aimed at establishing an evidence-base for co-designed interventions aimed at reducing chronic disease risk for First Nations peoples living on the islands of the Torres Strait. The broader project is situated within, and contributes to, a project already underway focusing on healthy ageing in the Torres Strait. Findings from this project will be incorporated into the wider Framework for Healthy Ageing being developed for the region. The research team also works with an existing Knowledge Circle (Indigenous Reference Group) that oversees the team's research in the region. The Knowledge Circle comprises Aboriginal and Torres Strait Islander community members, aged care workers, healthcare staff and academics who are committed to supporting the health of people in their communities. Projects are also conducted in partnership with local health and aged care providers in the Torres Strait.

ETHICS AND DISSEMINATION

This scoping review will map the assets that support health and wellbeing of Torres Strait Islanders living on the inhabited Islands of the Torres Strait. At a broader level, the findings will be of interest to

For peer review only - http://bmjopen.bmj.com/site/about/guidelines.xhtml

Torres Strait Islanders and policymakers. They could potentially be used to advocate for more and/or appropriate assets to support health and wellbeing across the Torres Strait. At the level of the broader project, the findings of the scoping review will be used to support the second phase. The second phase of the project is to yarn with community members about strengths and barriers to health and wellbeing that are part of their lives living on a Torres Strait Island. Phase two of this study will be led by Torres Strait Islander members of the research team with findings disseminated using a range of strategies including ongoing community engagement, conference presentations and publications.

Finally, as this is a desk-based review of publicly available sources ethical clearance is not necessary for this study. However, ethical clearance for the broader project has been obtained from the Far North Queensland Human Research Ethics Committee (reference HREC/2022/QCH/88155-1624).

Acknowledgements Janet Catterall Subject-specific Liaison Librarian.

Author contributions TW and KM designed the protocol and drafted the manuscript. TW, KM, MK, VW, SR, RQ, and ES reviewed and approved the final manuscript.

Funding This work was supported by [Australian National Health and Medical Research Council] grant number [MRF2016931].

Competing interests None declared

Patient and public involvement Patients and/or the public were involved in the design, or conduct, or reporting, or dissemination plans of this research. Refer to the Methods section for further details.

Patient consent for publication Not applicable.

Provenance and peer review. Not commissioned, externally peer reviewed.

For peer review only

ORCID ID Kathryn Meldrum https://orcid.org/ 0000-0003-1846-1596, Sarah Russell https://orcid.org/0000-0003-2845-5990, Rachel Quigley https://orcid.org/0000-0002-9943-9384 Edward Strivens https://orcid.org/0000-0002-7260-508X

For peer review only

For peer review only - http://bmjopen.bmj.com/site/about/guidelines.xhtml

REFERENCES

1. Melody SM, Bennett E, Clifford HD, Johnston FH, Shepherd CCJ, Alach Z, et al. A cross-sectional survey of environmental health in remote Aboriginal communities in Western Australia. *Int J Environ Health Res*. 2016;26(5-6):525-35.
2. Daniel M, Lekkas P, Cargo M, Stankov I, Brown A. Environmental risk conditions and pathways to cardiometabolic diseases in indigenous populations. *Annu Rev Public Health*. 2011;32:327-47.
3. Chakraborty A, Daniel M, Howard NJ, Chong A, Slavin N, Brown A, et al. Identifying Environmental Determinants Relevant to Health and Wellbeing in Remote Australian Indigenous Communities: A Scoping Review of Grey Literature. *Int J Environ Res Public Health*. 2021;18(8):4167.
4. Rose G. Sick individuals and sick populations. *Int J Epidemiol*. 1985;30(3):427-32.
5. Olvera Alvarez HA, Appleton AA, Fuller CH, Belcourt A, Kubzansky LD. An integrated socio-environmental model of health and well-being: A conceptual framework exploring the joint contribution of environmental and social exposures to health and disease over the life span. *Curr Environ Health Rep*. 2018;5(2):233-43.
6. Townsend M, Phillips R, Aldous D. "If the land is healthy… it makes the people healthy": The relationship between caring for Country and health for the Yorta Yorta Nation, Boonwurrung and Bangerang Tribes. *Health Place*. 2009;15(1):291-9.
7. Butler TL, Anderson K, Garvey G, Cunningham J, Ratcliffe J, Tong A, et al. Aboriginal and Torres Strait islander people's domains of wellbeing: a comprehensive literature review. *Soc Sci Med*. 2019;233:138-57.
8. Le Gal C, Dale MJ, Cargo M, Daniel M. Built environments and cardiometabolic morbidity and mortality in remote Indigenous communities in the Northern Territory, Australia. *Int J Environ Res Public Health*. 2020;17(3).
9. Marks E, Cargo MD, Daniel M. Constructing a health and social indicator framework for indigenous community health research. *Soc Indic Res*. 2007;82:93-110.
10. Daniel M, Cargo M, Marks E, Paquet C, Simmons D, Williams M, et al. Rating health and social indicators for use with indigenous communities: A tool for balancing cultural and scientific utility. *Soc Indic Res*. 2009;94:241-56.
11. Teasdale RM. Evaluative criteria: An integrated model of domains and sources. *Am J Eval*. 2021;42(3):354-76.
12. Centers for Disease Control and Prevention Program Performance and Evaluation Office. Indicators 2021 [updated 2022-01-31T07:35:53Z. Available from: https://www.cdc.gov/evaluation/indicators/index.htm.
13. Australian Institute of Health and Welfare (AIHW). Australia's health 2016. Canberra: Australian Institute of Health and Welfare; 2016. Cat no. AUS 199.
14. Gabi Titui Centre. Torres Strait n.d. [Available from: https://www.gabtitui.gov.au/torres-strait].
15. Australian Institute of Health and Welfare. Torres Strait Indigenous Region Dashboard for Health Conditions: @aihw; 2022 [Available from: https://www.rific.gov.au/regional-overview/dashboard.
16. Peters MDJ, Godfrey C, McInerney P, Munn Z, Tricco AC, Khalil H. Chapter 11: scoping reviews (2020 version). *JBI Manual for Evidence Synthesis*, JBI. 2020.
17. Peters MDJ, Marnie C, Tricco AC, Pollock D, Munn Z, Alexander L, et al. Updated methodological guidance for the conduct of scoping reviews. *JBI Evid Implement*. 2021;19(1):3-10.
18. The Endnote Team. Endnote. 20 ed. Philedelphia, PA: Clarivate; 2013.
19. Peters MDJ. Managing and coding references for systematic reviews and scoping reviews in EndNote. *Med Ref Serv Q*. 2017;36(1):19-31.
20. Preferred Reporting Items for Systematic reviews and Meta-analyses (PRISMA). Preferred Reporting Items for Systematic Reviews and Meta-analyses extension for scoping review (PRISMA-

For peer review only - http://bmjopen.bmj.com/site/about/guidelines.xhtml

ScR) flow diagram 2020 [Available from: http://www.prisma-statement.org/PRISMAStatement/FlowDiagram].

Figure legend

Figure 1. The Indigenous Indicator Classification System using an example from the built and natural environment subject group.

For peer review only

For peer review only - http://bmjopen.bmj.com/site/about/guidelines.xhtml

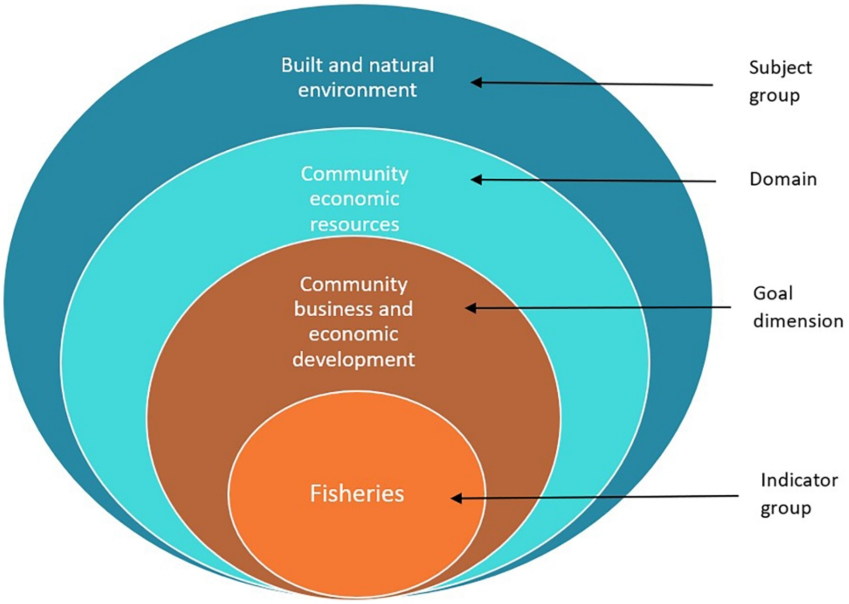

Figure 1. The Indigenous Indicator Classification System using an example from the built and natural environment subject group.

771x482mm (72 x 72 DPI)

Appendix A

Draft search strategy

14 April 2023

Informit

Databases (Health Collection, Humanities and Social Science Collection; Indigenous Collection; Aboriginal and Torres Strait Islander Health Bibliography (ATSIHealth); Indigenous Studies Bibliography (AIATSIS)

Torres Strait Island* AND community AND Environment AND (plan OR policy OR report) (all fields)

For peer review only

For peer review only - http://bmjopen.bmj.com/site/about/guidelines.xhtml

PRISMA-P (Preferred Reporting Items for Systematic review and Meta-Analysis Protocols) 2015 checklist: recommended items to address in a systematic review protocol*

Section and topic	Item No	Checklist item	Page
**ADMINISTRATIVE INFORMATION**			
Title:			
Identification	1a	Identify the report as a protocol of a systematic review	1
Update	1b	If the protocol is for an update of a previous systematic review, identify as such	n/a
Registration	2	If registered, provide the name of the registry (such as PROSPERO) and registration number	7
Authors:			
Contact	3a	Provide name, institutional affiliation, e-mail address of all protocol authors; provide physical mailing address of corresponding author	1
Contributions	3b	Describe contributions of protocol authors and identify the guarantor of the review	11
Amendments	4	If the protocol represents an amendment of a previously completed or published protocol, identify as such and list changes; otherwise, state plan for documenting important protocol amendments	n/a
Support:			
Sources	5a	Indicate sources of financial or other support for the review	11
Sponsor	5b	Provide name for the review funder and/or sponsor	11
Role of sponsor or funder	5c	Describe roles of funder(s), sponsor(s), and/or institution(s), if any, in developing the protocol	11
**INTRODUCTION**			
Rationale	6	Describe the rationale for the review in the context of what is already known	5
Objectives	7	Provide an explicit statement of the question(s) the review will address with reference to participants, interventions, comparators, and outcomes (PICO) Using Participant Concept Context (PCC) for Scoping review.	5
**METHODS**			
Eligibility criteria	8	Specify the study characteristics (such as PICO, study design, setting, time frame) and report characteristics (such as years considered, language, publication status) to be used as criteria for eligibility for the review	6
Information sources	9	Describe all intended information sources (such as electronic databases, contact with study authors, trial registers or other grey literature sources) with planned dates of coverage	7
Search strategy	10	Present draft of search strategy to be used for at least one electronic database, including planned limits, such that it could be repeated	7 & 14

For peer review only

Study records:			
Data management	11a	Describe the mechanism(s) that will be used to manage records and data throughout the review	9
Selection process	11b	State the process that will be used for selecting studies (such as two independent reviewers) through each phase of the review (that is, screening, eligibility and inclusion in meta-analysis)	8-9
Data collection process	11c	Describe planned method of extracting data from reports (such as piloting forms, done independently, in duplicate), any processes for obtaining and confirming data from investigators	8-9
Data items	12	List and define all variables for which data will be sought (such as PICO items, funding sources), any pre-planned data assumptions and simplifications Note – include example of database?	6, 9
Outcomes and prioritization	13	List and define all outcomes for which data will be sought, including prioritization of main and additional outcomes, with rationale	N/A
Risk of bias in individual studies	14	Describe anticipated methods for assessing risk of bias of individual studies, including whether this will be done at the outcome or study level, or both; state how this information will be used in data synthesis	N/A
Data synthesis	15a	Describe criteria under which study data will be quantitatively synthesised	9
	15b	If data are appropriate for quantitative synthesis, describe planned summary measures, methods of handling data and methods of combining data from studies, including any planned exploration of consistency (such as I^2, Kendall's τ)	N/A
	15c	Describe any proposed additional analyses (such as sensitivity or subgroup analyses, meta-regression)	N/A
	15d	If quantitative synthesis is not appropriate, describe the type of summary planned	N/A
Meta-bias(es)	16	Specify any planned assessment of meta-bias(es) (such as publication bias across studies, selective reporting within studies)	N/A
Confidence in cumulative evidence	17	Describe how the strength of the body of evidence will be assessed (such as GRADE)	N/A

*** It is strongly recommended that this checklist be read in conjunction with the PRISMA-P Explanation and Elaboration (cite when available) for important clarification on the items. Amendments to a review protocol should be tracked and dated. The copyright for PRISMA-P (including checklist) is held by the PRISMA-P Group and is distributed under a Creative Commons Attribution Licence 4.0.**

From: Shamseer L, Moher D, Clarke M, Ghersi D, Liberati A, Petticrew M, Shekelle P, Stewart L, PRISMA-P Group. Preferred reporting items for systematic review and meta-analysis protocols (PRISMA-P) 2015: elaboration and explanation. BMJ. 2015 Jan 2;349(jan02 1):g7647.

For peer review only

BMJ Open

Cultural, socio-political, environmental, and built assets supporting health and wellbeing in Torres Strait Island communities: Protocol for a scoping review

Journal:	*BMJ Open*
Manuscript ID	bmjopen-2023-077229.R1
Article Type:	Protocol
Date Submitted by the Author:	02-Nov-2023
Complete List of Authors:	Webb, Torres; James Cook University College of Medicine and Dentistry<br>Meldrum, Kathryn; James Cook University College of Medicine and Dentistry<br>Kilburn, Melissa; James Cook University College of Medicine and Dentistry<br>Wallace, Valda; James Cook University College of Medicine and Dentistry, College of Medicine and Dentistry<br>Russell, Sarah; James Cook University College of Medicine and Dentistry, College of Medicine and Dentistry<br>Quigley, Rachel; James Cook University College of Medicine and Dentistry, College of Medicine and Dentistry<br>Strivens, Edward; James Cook University College of Medicine and Dentistry; Queensland Health, Cairns and Hinterland Hospital and Health Service
**Primary Subject Heading**:	Public health
Secondary Subject Heading:	Occupational and environmental medicine
Keywords:	PUBLIC HEALTH, Risk Factors, Systematic Review, Geographical mapping < TROPICAL MEDICINE

SCHOLARONE™
Manuscripts

Cultural, socio-political, environmental, and built assets supporting health and wellbeing in Torres Strait Island communities: Protocol for a scoping review

Torres Webb, College of Medicine and Dentistry and Australian Institute of Health and Tropical Medicine, Building E1.003a, James Cook University, PO Box 6811, Cairns, Queensland, Australia. [1.]*

Kathryn Meldrum, College of Medicine and Dentistry and Australian Institute of Health and Tropical Medicine. Kathryn.meldrum@jcu.edu.au (corresponding author)*

Melissa Kilburn, College of Medicine and Dentistry and Australian Institute of Health and Tropical Medicine, James Cook University, Cairns, Queensland, Australia

Valda Wallace, College of Medicine and Dentistry and Australian Institute of Health and Tropical Medicine, James Cook University, Cairns, Queensland, Australia.[1.]

Sarah Russell, College of Medicine and Dentistry and Australian Institute of Health and Tropical Medicine, James Cook University, Cairns, Queensland, Australia.

Rachel Quigley, College of Medicine and Dentistry and Australian Institute of Health and Tropical Medicine, James Cook University, Cairns, Queensland, Australia.

Edward Strivens, College of Medicine and Dentistry and Australian Institute of Health and Tropical Medicine, James Cook University, Cairns, Queensland, Australia. Queensland Health, Cairns and Hinterland Hospital and Health Service, Cairns, Queensland, Australia.

[1.]This person identifies as a First Nations Australian

*Joint first authors

Word count - 2310

For peer review only - http://bmjopen.bmj.com/site/about/guidelines.xhtml

ABSTRACT

Introduction Risks to an individual's health should be considered alongside the environmental, socio-cultural and socio-political context(s) in which they live. Environmental mapping is an approach to identifying enablers and barriers to health within a community. The Indigenous Indicator Classification System (IICS) framework has been used to map the environment in Australian Indigenous communities. The IICS is a four-level nested hierarchical framework with subject groups including culture, socio-political and built at the top of the hierarchy and indicators at the bottom. The objective of this scoping review is to map the cultural, socio-political, environmental, and built assets that support health and wellbeing that exist in each Torres Strait Island community.

Methods and analysis This review will be conducted according the JBI method for scoping reviews. It will include sources that identify cultural, socio-political, environmental, and built assets that support health and wellbeing that exist in each Torres Strait Island community. Databases to be searched include: Informit; Scopus; Web of Science; HealthInfoNet, BioOne Complete and Green File. Sources of unpublished and grey literature will be located using Google and Google Scholar. Searches will be limited to the English language and literature published since January 2018 to ensure that the assets mapped reflect current conditions on each island. Data that answers the research question will be extracted from sources and recorded in an adaptation of the IICS. Quantitative analysis of the data will include summing each asset for individual islands and their associated clusters. Data will be presented graphically, diagrammatically, or in tabular form depending on what approach best conveys its meaning.

Ethics and dissemination The Far North Queensland Human Research Ethics Committee (reference HREC/2022/QCH/88155-1624) has approved this study. Dissemination of the review's findings will be led by Torres Strait Islander members of the research team through conferences and peer-reviewed publications.

For peer review only - http://bmjopen.bmj.com/site/about/guidelines.xhtml

STRENGTHS AND LIMITATIONS OF THIS STUDY

- The registration and publication of the protocol insures transparency of the review process.
- The scoping review will be conducted according to the method prescribed by the Joanna Briggs Institute.
- The Indigenous Indicator Classification System (IICS) used to identify and classify assets has been validated.
- Data collection will be limited to sources that are able to be located through desk-based searches which may mean that some cultural, socio-political, environmental, and built assets will not be recorded.

INTRODUCTION

The World Health Organisation estimates that 24% of all global deaths are linked with the environment [1]. Consequently, understanding the interactions between the environment and health, especially those exacerbated by climate change, can assist with managing their impact. Mapping the environment is an approach to identifying barriers to health and wellbeing within a community [2-4]. Environmental mapping is a robust approach because, as Rose [5] identified, the risk to an individual's health should be considered alongside the environmental, socio-cultural and socio-political context(s) in which they live. One approach to environmental mapping in an Australian Indigenous community in Western Australia focused mainly on barriers to health attributable to the environment [2]. This focus failed to recognise that the environment can also be a protective factor [6], especially in Australian Indigenous communities [3, 7, 8]. Le Gal and colleagues [9] supported a broader conceptualisation of health, inclusive of the environment, for Indigenous Australians. The rationale for broader conceptualisation builds on the previous work of Daniel and his colleagues [3]. Their review [3] argued that the context and composition of the environment act together as risks for health and wellbeing in Australian Indigenous communities. The authors defined context as the "properties of places" (p. 330) and composition as "collective attributes of people in a place" (p. 330) [3].

For peer review only - http://bmjopen.bmj.com/site/about/guidelines.xhtml

Environmental context and composition coalesce to interact with an individual's genetic, behavioural and stress axes to predispose them to chronic disease.

The Indigenous Indicator Classification System as an approach to environmental mapping

Early work by Marks and colleagues [10, 11] proposed the Indigenous Indicator Classification System (IICS) as a framework that could be used to map the "distinct historical, social and cultural contexts of Indigenous communities" (p. 93). The IICS was developed in 2006 with input from Indigenous peoples from Canada, Aotearoa/New Zealand and Australia [3] and validated in 2009 [11].

The IICS is a hierarchical four-level system with subject groups at the highest level. At the time of development, subject groups consisted of culture, socio-demographic, socio-political, socio-economic, built, and natural environment, psychosocial, and social organisations. For example, cultural assets include the background and history of peoples and the traditional activities and cultural responsibilities that they undertake [10]. Socio-political assets include, but are not limited to, community economic resources, Indigenous self-government and autonomy and labour market and working conditions. Whereas, built assets include but are not limited to the natural environment, housing, and transportation. The subject groups are the broadest classification level to which each indicator group or goal dimension is allocated to [10]. Domains are the broadest of the nested hierarchical categories [10] and identify the focus of the goal dimension and/or indicator group [12]. Goal dimensions describe the parameters for classifying indicators and could be used to identify potential targets for health policies and/or programs [10]. Consequently, in the IICS they often describe a desirable community outcome. For example, community business and economic development [4]. Indicators are measurable information [13] able to be found in documents, grey literature or through community conversations. In Australia, health indicators, such as those used in the IICS, describe particular elements of health or aspects of performance [14]. The link(s) between

For peer review only - http://bmjopen.bmj.com/site/about/guidelines.xhtml

domains and indicators are important for explaining and providing a platform for investigating the complexity of influences on Indigenous peoples' health [10] and wellbeing. Figure 1 below outlines the IICS. Figure 2 illustrates the IICS using two examples, the built and natural environment and socio-political.

Insert Figures 1 and 2 here

The context of the protocol and scoping review

The setting for this study is the Torres Strait (Zenadth Kes) situated between the tip of the eastern Australian mainland and Papua New Guinea. There are over 200 islands in the Torres Strait located in a vast geographic area of over 44,000 square kilometres. However, only 17 islands are permanently inhabited [15]. In 2016, approximately 7403 people lived on the islands of the Torres Strait, 86.6% of whom identified as Australian First Nations peoples [16].

Rationale and aims

The context of this scoping review is a broader project funded by the Australian National Health and Medical Research Council (grant number MRF2016931) designed to address significant gaps in existing knowledge and develop an understanding of key assets for a strengths-based approach to targeting diet and activity components of chronic disease risk for First Nations peoples living in the Torres Strait. The broader project is designed to:

- Identify the strengths and barriers to good health in Torres Strait communities;
- Identify dietary and activity behaviours of Australian First Nations peoples living in the Torres Strait; and,
- Use this information to develop community-led chronic disease prevention interventions at an individual and service provider level.

For peer review only

The aim of the scoping review that this protocol refers to is to addresses dot point 1) (above) initially by mapping the cultural, socio-political, natural, and built environment of each of the inhabited Islands of the Torres Strait. The research question guiding this scoping review is: What cultural, socio-political, environmental, and built assets that support health and wellbeing exist in each Torres Strait Island community?

METHODS AND ANALYSIS

The proposed scoping review will be conducted in accordance with the JBI methodology for scoping reviews [17]. Both this protocol and subsequent scoping review will be reported using the Preferred Reporting Items for Systematic Reviews and Meta-analyses Extension for Scoping Review (PRISMA-ScR) guidelines [18].

Inclusion criteria

In line with the JBI methodology the Participant, Concept, Context (PCC) framework will be used to guide inclusion criteria and search strategy for the scoping review.

Participants

The scoping review will include sources that identify and/or describe any cultural, socio-political, natural, and built assets (hereafter referred to collectively as assets) that support health and wellbeing in Torres Strait Island communities. Assets may include people and programs.

Concept

The concepts to be examined in the scoping review will include assets that support health and wellbeing in Torres Strait Island communities. Specific data to be extracted includes:

- The island and/or community where the asset is located;
- The type of asset.

For peer review only - http://bmjopen.bmj.com/site/about/guidelines.xhtml

Sources excluded from the data set includes any assets identified from a secondary source. For example, data in published literature must come from research conducted by the authors. The rationale for excluding secondary sources is that it may be out of date or may not be accurate. In addition, the information must describe actual assets not aspirational ones that may be detailed in regional plans.

Context

Any source that details the assets of Torres Strait Island communities will be considered for inclusion in the scoping review. The sources may be obtained from database searches or from grey literature located on key regional governance websites such as Torres Strait Regional Authority, Torres Strait Island Regional Council, Gur A Baradharaw Kod Torres Strait Sea and Land Council, and Torres Shire Council.

Types of sources

Relevant sources that have been peer-reviewed or have been obtained from grey literature will be included in the data set. Sources may include annual reports, strategic, corporate, operational or implementation plans, evaluations, planning documents and published literature. Critical reviews, literature reviews and systematic reviews will not be included in the data set because they are not primary data sources. However, their reference lists will be reviewed for relevant primary data sources.

Inclusion criteria in order of priority will be:

1. Information that identifies assets available/present in a Torres Strait Island community.
2. Uses primary data sources.
3. Has been published since 2018.

The scoping review is registered with The Open Science Framework (https://osf.io/2wv8c).

For peer review only - http://bmjopen.bmj.com/site/about/guidelines.xhtml

Search strategy

The search strategy will aim to locate published and unpublished sources. A three-step search strategy will be utilised in this review. First, an initial limited search of Web of Science and Informit was undertaken to identify relevant literature. The text words contained in the titles and abstracts of relevant sources, and the index terms used to describe them were used to develop a full search strategy tailored to each information source. For example, a pilot search strategy is illustrated in the online supplement materials (*Appendix A*). Step one was supported by a subject-specific liaison librarian. The reference lists of all included sources of evidence will be screened for additional sources. Additionally, reference lists of systematic reviews on the same or similar topic will be scrutinised for appropriate papers, reports, or other data sources.

Published or unpublished reports or other data sources in the English language and available since the year 2018 to present will be included in the data set. Sources published since 2018 will be used because they are more likely to identify assets currently present in Torres Strait Island communities.

Databases to be searched include:

- Informit;
- Scopus;
- Web of Science;
- BioOne Complete;
- HealthInfoNet;
- Green File.

Unpublished and grey literature will be identified using Google, Google Scholar and website searches. Google and Google Scholar searches will use the search string Torres Strait Island* AND community AND environment AND plan OR Policy OR report with a date limiter from 1 Jan 2018 – date of search.

For peer review only - http://bmjopen.bmj.com/site/about/guidelines.xhtml

Source of evidence selection

At the commencement of step two all identified citations will be collated and uploaded into EndNote (Version 20.1) [19] and data will be subsequently managed according to the method proposed by Peters [20]. A pilot test will then be conducted as described by Peters and colleagues [17], following which, titles and abstracts will be screened by two independent reviewers for assessment against the inclusion criteria for the review. Potentially relevant sources will be retrieved in full and subsequently assessed against the inclusion criteria by two independent reviewers. Reasons for exclusion of full text sources of evidence that do not meet the inclusion criteria will be recorded and reported in the scoping review. Any disagreements that arise between the reviewers at each stage of the selection process will be resolved through discussion. Where agreement cannot be achieved through discussion an additional reviewer/s will review the source. The results of the search and the study inclusion process will be reported in full in the final scoping review and presented in a Preferred Reporting Items for Systematic Reviews and Meta-analyses extension for scoping review (PRISMA-ScR) flow diagram [21].

Data extraction

Before data extraction, the IICS will be adapted for the context of this study by the co-fist authors (TW, KM). Subsequently, data will be manually extracted from sources included in the scoping review by two independent reviewers using a database adaptation of the IICS [4, 10, 11]. Data extracted from sources will include specific information about the participants, concept, and context. Any further adaptations to the IICS made during data extraction will be detailed in the scoping review. Any disagreements will be managed in the manner previously discussed. If appropriate, source authors will be contacted to request missing or additional information, where required. In addition, key informants in regional governance bodies will be contacted to confirm source information when necessary.

Data analysis and presentation

For peer review only - http://bmjopen.bmj.com/site/about/guidelines.xhtml

Data will be analysed quantitatively to identify how it is distributed geographically across the Torres Strait Islands. Assets used will be categorised into the overarching groups:

- Cultural;
- Socio-political;
- Environmental, and,
- Built.

Once an asset has been categorised into a group it will subsequently be allocated into an indicator group by referring to the relevant indicator statement. The presence of an asset will be recorded by typing a '1' into the relevant column in the database. Once data collection is complete, asset sums will be calculated for each indicator, for each Island and Island group.

Data will be presented graphically, diagrammatically or in tabular form depending on what approach that best represents the data. For example, using Geographic Information System (GIS) mapping software may be used to indicate what assets are available on each island as this approach potentially conveys greater meaning than tabulating it. A narrative summary will accompany the tabulated and/or charted results and will describe how the results relate to the review's objective and question.

Patient and public involvement

As previously indicated this study is phase one of a broader four phase study aimed at establishing an evidence-base for co-designed interventions aimed at reducing chronic disease risk for First Nations peoples living on the islands of the Torres Strait. The broader project is situated within, and contributes to, a project already underway focusing on healthy ageing in the Torres Strait. Findings from this project will be incorporated into the wider Framework for Healthy Ageing being developed for the region. The research team also works with an existing Knowledge Circle (Indigenous Reference Group) that oversees the team's research in the region. The Knowledge Circle comprises Aboriginal

For peer review only - http://bmjopen.bmj.com/site/about/guidelines.xhtml

For peer review only

and Torres Strait Islander community members, aged care workers, healthcare staff and academics who are committed to supporting the health of people in their communities. Projects are also conducted in partnership with local health and aged care providers in the Torres Strait.

ETHICS AND DISSEMINATION

This scoping review will map the assets that support health and wellbeing of Torres Strait Islanders living on the inhabited Islands of the Torres Strait. At a broader level, the findings will be of interest to Torres Strait Islanders and policymakers. They could potentially be used to advocate for more and/or appropriate assets to support health and wellbeing across the Torres Strait. At the level of the broader project, the findings of the scoping review will be used to support the second phase. The second phase of the project is to yarn with community members about strengths and barriers to health and wellbeing that are part of their lives living on a Torres Strait Island. Phase two of this study will be led by Torres Strait Islander members of the research team with findings disseminated using a range of strategies including ongoing community engagement, conference presentations and publications.

Finally, as this is a desk-based review of publicly available sources ethical clearance is not necessary for this study. However, ethical clearance for the broader project has been obtained from the Far North Queensland Human Research Ethics Committee (reference HREC/2022/QCH/88155-1624).

Acknowledgements Janet Catterall Subject-specific Liaison Librarian.

Author contributions TW and KM designed the protocol and drafted the manuscript. TW, KM, MK, VW, SR, RQ, and ES reviewed and approved the final manuscript.

Funding This work was supported by an Australian National Health and Medical Research Council grant number [MRF2016931].

Competing interests None declared

For peer review only

Patient and public involvement Patients and/or the public were involved in the design, or conduct, or reporting, or dissemination plans of this research. Refer to the Methods section for further details.

Patient consent for publication Not applicable.

Provenance and peer review. Not commissioned, externally peer reviewed.

ORCID ID Kathryn Meldrum https://orcid.org/ 0000-0003-1846-1596, Sarah Russell https://orcid.org/0000-0003-2845-5990, Rachel Quigley https://orcid.org/0000-0002-9943-9384 Edward Strivens https://orcid.org/0000-0002-7260-508X

For peer review only

REFERENCES

1. World Health Organisation. Public health and environment 2023 [23/10/2023]. Available from: https://www.who.int/data/gho/data/themes/public-health-and-environment.
2. Melody SM, Bennett E, Clifford HD, Johnston FH, Shepherd CCJ, Alach Z, et al. A cross-sectional survey of environmental health in remote Aboriginal communities in Western Australia. *Int J Environ Health Res*. 2016;26(5-6):525-35.
3. Daniel M, Lekkas P, Cargo M, Stankov I, Brown A. Environmental risk conditions and pathways to cardiometabolic diseases in indigenous populations. *Annu Rev Public Health*. 2011;32:327-47.
4. Chakraborty A, Daniel M, Howard NJ, Chong A, Slavin N, Brown A, et al. Identifying Environmental Determinants Relevant to Health and Wellbeing in Remote Australian Indigenous Communities: A Scoping Review of Grey Literature. *Int J Environ Res Public Health*. 2021;18(8):4167.
5. Rose G. Sick individuals and sick populations. *Int J Epidemiol*. 1985;30(3):427-32.
6. Olvera Alvarez HA, Appleton AA, Fuller CH, Belcourt A, Kubzansky LD. An integrated socio-environmental model of health and well-being: A conceptual framework exploring the joint contribution of environmental and social exposures to health and disease over the life span. *Curr Environ Health Rep*. 2018;5(2):233-43.
7. Townsend M, Phillips R, Aldous D. "If the land is healthy… it makes the people healthy": The relationship between caring for Country and health for the Yorta Yorta Nation, Boonwurrung and Bangerang Tribes. *Health Place*. 2009;15(1):291-9.
8. Butler TL, Anderson K, Garvey G, Cunningham J, Ratcliffe J, Tong A, et al. Aboriginal and Torres Strait islander people's domains of wellbeing: a comprehensive literature review. *Soc Sci Med*. 2019;233:138-57.
9. Le Gal C, Dale MJ, Cargo M, Daniel M. Built environments and cardiometabolic morbidity and mortality in remote Indigenous communities in the Northern Territory, Australia. *Int J Environ Res Public Health*. 2020;17(3).
10. Marks E, Cargo MD, Daniel M. Constructing a health and social indicator framework for indigenous community health research. *Soc Indic Res*. 2007;82:93-110.
11. Daniel M, Cargo M, Marks E, Paquet C, Simmons D, Williams M, et al. Rating health and social indicators for use with indigenous communities: A tool for balancing cultural and scientific utility. *Soc Indic Res*. 2009;94:241-56.
12. Teasdale RM. Evaluative criteria: An integrated model of domains and sources. *Am J Eval*. 2021;42(3):354-76.
13. Centers for Disease Control and Prevention Program Performance and Evaluation Office. Indicators 2021 [updated 2022-01-31T07:35:53Z. Available from: https://www.cdc.gov/evaluation/indicators/index.htm.
14. Australian Institute of Health and Welfare (AIHW). Australia's health 2016. Canberra: Australian Institute of Health and Welfare; 2016. Cat no. AUS 199.
15. Gabi Titui Centre. Torres Strait n.d. [Available from: https://www.gabtitui.gov.au/torres-strait].
16. Australian Institute of Health and Welfare. Torres Strait Indigenous Region Dashboard for Health Conditions: @aihw; 2022 [Available from: https://www.rific.gov.au/regional-overview/dashboard.
17. Peters MDJ, Godfrey C, McInerney P, Munn Z, Tricco AC, Khalil H. Chapter 11: scoping reviews (2020 version). *JBI Manual for Evidence Synthesis*, JBI. 2020.
18. Peters MDJ, Marnie C, Tricco AC, Pollock D, Munn Z, Alexander L, et al. Updated methodological guidance for the conduct of scoping reviews. *JBI Evid Implement*. 2021;19(1):3-10.
19. The Endnote Team. Endnote. 20 ed. Philedelphia, PA: Clarivate; 2013.
20. Peters MDJ. Managing and coding references for systematic reviews and scoping reviews in EndNote. *Med Ref Serv Q*. 2017;36(1):19-31.

For peer review only - http://bmjopen.bmj.com/site/about/guidelines.xhtml

21. Preferred Reporting Items for Systematic reviews and Meta-analyses (PRISMA). Preferred Reporting Items for Systematic Reviews and Meta-analyses extension for scoping review (PRISMA-ScR) flow diagram 2020 [Available from: http://www.prisma-statement.org/PRISMAStatement/FlowDiagram].

Figure legend

Figure 1. The Indigenous Indicator Classification System [adapted from 2.]

Figure 2. Examples of the Indigenous Indicator Classification System.

For peer review only

For peer review only - http://bmjopen.bmj.com/site/about/guidelines.xhtml

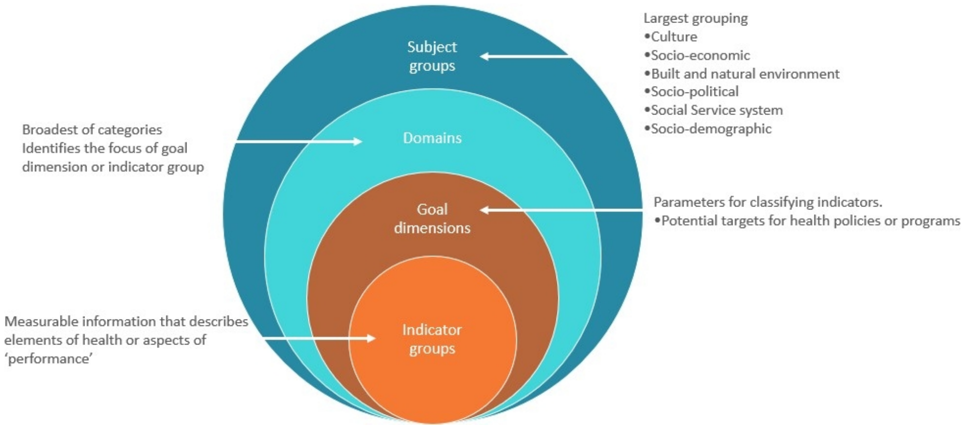

Figure 1 - The Indigenous Indicator Classification System [adapted from 2.]

269x118mm (96 x 96 DPI)

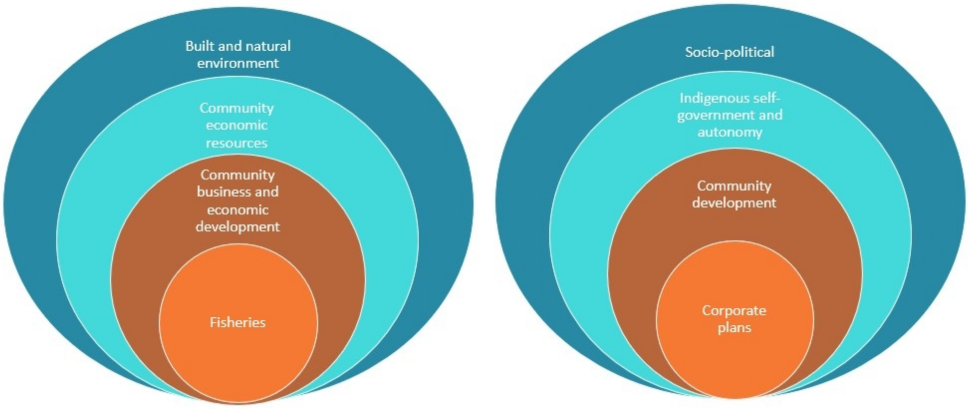

Figure 2 - Examples of the Indigenous Indicator Classification System.

265x114mm (96 x 96 DPI)

Appendix A

Draft search strategy

14 April 2023

Informit

Databases (Health Collection, Humanities and Social Science Collection; Indigenous Collection; Aboriginal and Torres Strait Islander Health Bibliography (ATSIHealth); Indigenous Studies Bibliography (AIATSIS)

Torres Strait Island* AND community AND Environment AND (plan OR policy OR report) (all fields)

For peer review only

For peer review only - http://bmjopen.bmj.com/site/about/guidelines.xhtml

PRISMA-P (Preferred Reporting Items for Systematic review and Meta-Analysis Protocols) 2015 checklist: recommended items to address in a systematic review protocol*

Section and topic	Item No	Checklist item	Page
**ADMINISTRATIVE INFORMATION**			
Title:			
Identification	1a	Identify the report as a protocol of a systematic review	1
Update	1b	If the protocol is for an update of a previous systematic review, identify as such	n/a
Registration	2	If registered, provide the name of the registry (such as PROSPERO) and registration number	7
Authors:			
Contact	3a	Provide name, institutional affiliation, e-mail address of all protocol authors; provide physical mailing address of corresponding author	1
Contributions	3b	Describe contributions of protocol authors and identify the guarantor of the review	11
Amendments	4	If the protocol represents an amendment of a previously completed or published protocol, identify as such and list changes; otherwise, state plan for documenting important protocol amendments	n/a
Support:			
Sources	5a	Indicate sources of financial or other support for the review	11
Sponsor	5b	Provide name for the review funder and/or sponsor	11
Role of sponsor or funder	5c	Describe roles of funder(s), sponsor(s), and/or institution(s), if any, in developing the protocol	11
**INTRODUCTION**			
Rationale	6	Describe the rationale for the review in the context of what is already known	5
Objectives	7	Provide an explicit statement of the question(s) the review will address with reference to participants, interventions, comparators, and outcomes (PICO) Using Participant Concept Context (PCC) for Scoping review.	5
**METHODS**			
Eligibility criteria	8	Specify the study characteristics (such as PICO, study design, setting, time frame) and report characteristics (such as years considered, language, publication status) to be used as criteria for eligibility for the review	6
Information sources	9	Describe all intended information sources (such as electronic databases, contact with study authors, trial registers or other grey literature sources) with planned dates of coverage	7
Search strategy	10	Present draft of search strategy to be used for at least one electronic database, including planned limits, such that it could be repeated	7 & 14

For peer review only

Study records:			
Data management	11a	Describe the mechanism(s) that will be used to manage records and data throughout the review	9
Selection process	11b	State the process that will be used for selecting studies (such as two independent reviewers) through each phase of the review (that is, screening, eligibility and inclusion in meta-analysis)	8-9
Data collection process	11c	Describe planned method of extracting data from reports (such as piloting forms, done independently, in duplicate), any processes for obtaining and confirming data from investigators	8-9
Data items	12	List and define all variables for which data will be sought (such as PICO items, funding sources), any pre-planned data assumptions and simplifications Note – include example of database?	6, 9
Outcomes and prioritization	13	List and define all outcomes for which data will be sought, including prioritization of main and additional outcomes, with rationale	N/A
Risk of bias in individual studies	14	Describe anticipated methods for assessing risk of bias of individual studies, including whether this will be done at the outcome or study level, or both; state how this information will be used in data synthesis	N/A
Data synthesis	15a	Describe criteria under which study data will be quantitatively synthesised	9
	15b	If data are appropriate for quantitative synthesis, describe planned summary measures, methods of handling data and methods of combining data from studies, including any planned exploration of consistency (such as I^2, Kendall's τ)	N/A
	15c	Describe any proposed additional analyses (such as sensitivity or subgroup analyses, meta-regression)	N/A
	15d	If quantitative synthesis is not appropriate, describe the type of summary planned	N/A
Meta-bias(es)	16	Specify any planned assessment of meta-bias(es) (such as publication bias across studies, selective reporting within studies)	N/A
Confidence in cumulative evidence	17	Describe how the strength of the body of evidence will be assessed (such as GRADE)	N/A

*** It is strongly recommended that this checklist be read in conjunction with the PRISMA-P Explanation and Elaboration (cite when available) for important clarification on the items. Amendments to a review protocol should be tracked and dated. The copyright for PRISMA-P (including checklist) is held by the PRISMA-P Group and is distributed under a Creative Commons Attribution Licence 4.0.**

From: Shamseer L, Moher D, Clarke M, Ghersi D, Liberati A, Petticrew M, Shekelle P, Stewart L, PRISMA-P Group. Preferred reporting items for systematic review and meta-analysis protocols (PRISMA-P) 2015: elaboration and explanation. BMJ. 2015 Jan 2;349(jan02 1):g7647.

For peer review only
